# Integrating Biosensors in Organs-on-Chip Devices: A Perspective on Current Strategies to Monitor Microphysiological Systems

**DOI:** 10.3390/bios10090110

**Published:** 2020-08-28

**Authors:** Erika Ferrari, Cecilia Palma, Simone Vesentini, Paola Occhetta, Marco Rasponi

**Affiliations:** Department of Electronics, Information and Bioengineering, Politecnico di Milano, 20133 Milano, Italy; erika1.ferrari@polimi.it (E.F.); cecilia.palma@polimi.it (C.P.); simone.vesentini@polimi.it (S.V.); paola.occhetta@polimi.it (P.O.)

**Keywords:** organs-on-chip, microphysiological systems, microfluidics, microfabrication, biosensors, electrodes, multiorgan-on-chip, drug screening, in vitro models

## Abstract

Organs-on-chip (OoC), often referred to as microphysiological systems (MPS), are advanced in vitro tools able to replicate essential functions of human organs. Owing to their unprecedented ability to recapitulate key features of the native cellular environments, they represent promising tools for tissue engineering and drug screening applications. The achievement of proper functionalities within OoC is crucial; to this purpose, several parameters (e.g., chemical, physical) need to be assessed. Currently, most approaches rely on off-chip analysis and imaging techniques. However, the urgent demand for continuous, noninvasive, and real-time monitoring of tissue constructs requires the direct integration of biosensors. In this review, we focus on recent strategies to miniaturize and embed biosensing systems into organs-on-chip platforms. Biosensors for monitoring biological models with metabolic activities, models with tissue barrier functions, as well as models with electromechanical properties will be described and critically evaluated. In addition, multisensor integration within multiorgan platforms will be further reviewed and discussed.

## 1. Introduction

The development of microengineered in vitro models able to reproduce architecture and functionality of human organs and tissues is expected to boost the drug development field advancement. These models are promising tools for elucidating the biological mechanisms underlying morphogenetic and pathogenetic processes, as well as for investigating cellular mechanisms which are paramount for the improvement of drug screening process [1,2,3]. 

Two-dimensional (2D) cell cultures on Petri dishes or similar standard culture platforms are typically used in pharmaceutical research and development, as they offer multiple advantages, such as simplicity, low cost, and ease of handling. However, obtaining specific cell phenotypes and reproducing in vivo cell–cell, as well as cell–extracellular matrix (ECM), interactions is challenging in static cultures [4]. More recently, spheroid technology was introduced to develop organoids, i.e., in vitro tissue models derived from pluripotent stem cells or isolated organ progenitors that differentiate into multiple cell types and self-organize in a manner similar to in vivo [5]. Organoids thus allow to coculture multiple cell types in three-dimensional (3D) configurations and to recreate more physiologically relevant settings in vitro [6], but they show some disadvantages, such as relatively high costs, lack of reproducibility and of standardization protocols, as well as absence of vascularization and formation of necrotic cores [7,8]. In this scenario, organs-on-chip, OoC (also called microphysiological systems, MPS) are being investigated as an alternative to standard in vitro tools and potentially to animal models. OoC are miniaturized in vitro models building upon advanced 2D and 3D human cell cultures reproducing specific tissue architectures, supporting cell viability and organ functions. OoC have been proved able to predict human-specific clinical outcomes better than current preclinical models [9,10,11]. These platforms also provide a variety of advantages inherited from miniaturization, including fast and inexpensive fabrication techniques, design flexibility, as well as reduced consumption of reagents and precious cells (e.g., patient specific cells and induced pluripotent stem cells) [12,13,14]. OoCs are thus raising increasing expectation as promising tools for disease modeling, drug testing, and personalized medicine [15,16,17,18].

In details, OoCs are not intended to reproduce a whole living organ, but rather to establish the minimal functional unit able to recapitulate certain aspects of human physiology or pathophysiology in a controlled and straightforward manner [17,19]. Various organs and tissues have been modeled, spanning from lung [20,21], heart [22,23], liver [24,25], gut [26,27], kidney [28,29], muscle [30,31], bone [32,33], cartilage [34,35], blood-brain barrier [36,37], as well as nervous systems [38,39]. Many of these systems incorporate biophysical and/or biochemical stimuli (i.e., mechanical [22,34], electrical [40] and biochemical cues [41]) to mimic the 3D in vivo physiological environment of the corresponding native organ and to induce the proper cellular phenotypes and tissue maturation. However, recapitulating and monitoring in a single system the controlled and dynamic exchange of molecules and dissolved gases, the complicated tissue-tissue interactions, as well as the complex tissue pathophysiology is still an open challenge [42].

One of the major limitations of current OoC is the hurdle to collect real-time information about the cells and the surrounding environment in order to monitor online tissue maturation and response to stimuli (e.g., drug administration). Several characterization methods, such as viability assays and biomarker quantification to assess, respectively, cytotoxicity or functionality, still rely on off-chip analysis (i.e., ELISA technology) and imaging techniques for data acquisition and are mostly limited to end-point assays [43]. Major progresses in this field are related to the integration of biosensors for continuous, noninvasive, and on-line monitoring of parameters of interest. This, together with the possibility of parallelizing samples analysis characteristic of OoC, holds the promise to improve the exploitation of these in vitro systems as preclinical models and drug screening platforms [42,43]. Specifically, a range of electrical, magnetic, and optical biosensing approaches have been reported over the last years and previously reviewed [42,43,44,45] for monitoring cell populations within microfabricated OoC systems, with high sensitivity and high resolution [46,47,48,49]. For instance, biosensors for monitoring cell growth and behavior [50], electrical [51,52,53] and mechanical [30,54,55] properties, as well as environmental parameters such as oxygen [56,57,58], pH [59], and metabolites concentration [60,61] have been studied and validated [62].

In this review, we focus on the current strategies to miniaturize and embed biosensing systems into OoC platforms. Biosensors for monitoring biological models with metabolic activities (i.e., nervous, cardiac, intestinal, hepatic, pancreatic, renal, and immune systems), models with tissue barrier functions (i.e., brain, gut, lung, and skin), as well as models with electromechanical properties (i.e., nervous, muscular, and cardiac systems) will be described and critically evaluated. In addition, multisensor integration within multiorgan platforms will be further reviewed and discussed.

## 2. Biosensors for Measuring OoC’ Metabolic Activity

The major determinant for the formation of functional tissues in in vitro OoC systems is the cell culture environment where cells are exposed to changes of oxygen (O_2_) levels, pH, nutrient content as well as gradients of secreted metabolites (i.e., glucose and lactate) from adjacent cells. Integration of analytical biosensors to OoC platforms enable to achieve controllable and reproducible cell culture environments. Specifically, different microsensors have been integrated for monitoring the metabolic activities of cultured cells [1]. In the next paragraphs, we will describe OoC-integrated biosensors for measuring oxygen, glucose, lactate, and various types of cytokines.

### 2.1. Oxygen

Oxygen is a key parameter in cell metabolism as its concentration is crucial for both aerobic and anaerobic pathways in human tissues. In the human body, regions of low oxygen content (<5%), such as the brain and gut, exist in proximity to regions of much higher oxygen (>11%), such as the arterial blood [63]. Thanks to precise and continuous monitoring of oxygen levels in OoCs, the maintenance of such physiologically relevant oxygen distribution in tissues and organs can be achieved [1]. 

Two types of oxygen sensors have been developed for OoC applications, namely, optical and electrochemical. Optical sensors are based on fluorescence quenching in an appropriate dye by molecular oxygen [64] and are particularly appealing for OoC systems, whose typical optical transparency allows a fast integration enabling noninvasive measurements. On the other hand, the simplicity, high sensitivity, and easy miniaturization of electrochemical sensors (e.g., amperometric) make them robust and reproducible solutions for in situ O_2_ monitoring [65]. Amperometric oxygen biosensors consist of a sensor element which, upon selective recognition and interaction with oxygen molecules, produces an electrical signal linearly proportional to the oxygen concentration [66]. Although both sensor types have been widely used for a variety of applications, optical techniques are still preferred in case of low oxygen levels since electrochemical sensors consume oxygen during measurement [67].

Among all, mitochondrial function plays a pivotal role in facilitating cellular maturation and metabolism. When the oxygen level is reduced in aerobic culture environments, cells adapt to the consequent loss of mitochondrial respiration by activating anaerobic pathways (e.g., glycolysis and glutaminolysis [68]) and this eventually leads to undesirable stress in the culture. Along with culture microenvironment, other factors such as cell type, type of material used for the OoC fabrication, ECM-like coating, and perfusion conditions can influence oxygen availability in OoC models. For instance, Zirath et al. [56] studied the effects of cell size and surface coating on oxygen concentration in a microfluidic device. By adopting oxygen optical sensor spots, they found a higher oxygen demand in presence of either bigger cell types or cells cultured on ECM-like adhesion promoters. In another study, Rennert et al. [57] adopted a compartmentalized microfluidic device for liver cell culture to study the impact of the perfusion rate on oxygen consumption by HepaRG cells. Specifically, with luminescence-based sensors, they found that at low flow rates (i.e., 1 µL/min), the oxygen consumption by hepatocytes was faster than oxygen resupply through medium perfusion, whereas oxygen saturation (i.e., 95%) was reached with 3 µL/min of medium perfusion. Hence, real-time continuous on-line measurement of oxygen content may preliminarily reveal and prevent from mitochondrial dysfunctions, thus facilitating the optimization of culture conditions [69].

**Figure 1:**
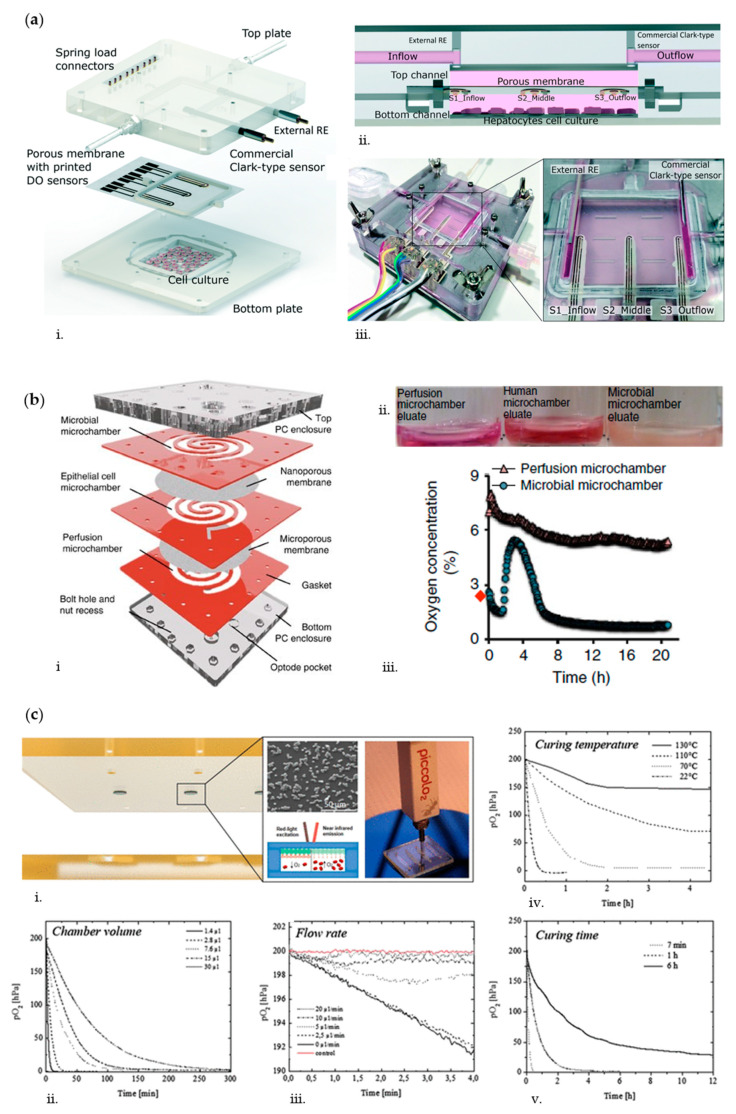
(**a**) Schematic of the compartments composing the ExoLiver (i); cross-section of the bioreactor showing the position of the three sensors (ii); real picture of the ExoLiver with all the fluidic and electrical connections (iii). Adapted from Ref. [58] with permission from The Royal Society of Chemistry. (**b**) Exploded representative view of the HuMiX device (ii): elastomeric gaskets (thickness: 700 μm) sandwiched between two polycarbonate enclosures, which host the optical oxygen sensors; each gasket defines a distinct spiral-shaped microchannel (length of 200 mm, width of 4 mm, and height of 0.5 mm); a microporous membrane (pore diameter of 1 μm), which allows diffusion-dominant perfusion to the human cells, is used to partition the perfusion and human microchambers, whereas a nanoporous membrane (pore diameter of 50 nm) partitions the human and microbial microchambers to prevent the infiltration of microorganisms into the human microchamber (i); sampled eluates from the HuMiX device following a 24 h coculture with the anaerobe *Lactobacillus rhamnosus* GG (LGG) (ii); oxygen concentration profiles within the perfusion and microbial microchambers upon initiation of the coculture with LGG. The red dot indicates the preinoculation oxygen concentration of 2.6% in the microbial microchamber (iii). Adapted from Ref. [75] with permission from Springer Nature. (**c**) Oxygen biosensing principle of the thiol–ene-epoxy biochip (i); 5 μm oxygen sensor microparticles attached to the reactive polymeric surface of the thiol-ene-epoxy biochip; tuning of oxygen scavenging of thiol-ene-epoxy biochips with varying (ii) microchannel volumes of 1.4 μL (h = 45 μm), 2.8 μL (h = 90 μm), 7.6 μL (h = 250 μm), 15 μL (h = 500 μm), and 30 μL (h = 750 μm), (iii) flow rates in the range of 0−20 μL/min, (iv) curing temperature in the range of 22−130 °C (h = 90 μm), and (v) curing time in the range of 7 min to 6 h (h = 90 μm, 150 °C curing temperature). Adapted from Ref. [78] with permission from ACS Publications.

Overall, oxygen monitoring seems particularly relevant for liver and gut applications. In the native liver, the oxygen gradient that develops along the liver sinusoid induces a demarcation of function, termed metabolic zonation, which directly affects nutrient metabolism, morphology, and xenobiotic transformation in hepatocytes [70]. Bavli et al. [69] developed a liver-on-chip based on a polymethyl methacrylate (PMMA) bioreactor in which polydimethylsiloxane (PDMS) microwell inserts could be perfused under physiological conditions (2 μL/min) to mimic such liver zonation. In particular, they cultured HepG2/C3A cells spheroids for 28 days together with probes enabling to monitor mitochondrial respiration in real-time. Specifically, these probes were composed of 50 μm diameter polystyrene microbeads loaded with a ruthenium-based phosphorescence dye that showed a decrease in phosphorescence decay time as a function of oxygen concentration. To assess the functionality of such optical oxygen sensor, they challenged the spheroids with rotenone (1, 50, and 200 µM), an organic pesticide known to induce apoptosis at low concentrations [71], and troglitazone (50–2000 µM), an anti-inflammatory drug that was removed from market due to severe drug-induced liver toxicity [72]. With both compounds, oxygen uptake dropped rapidly following a dose-dependent cellular damage. In another study, Moya et al. [58] developed a modular bioreactor (ExoLiver, Figure 1a) composed of an upper microfluidic chamber and a lower static chamber for culturing primary human hepatocytes (PHH). The two chambers were separated by a polytetrafluoroethylene (PTFE) membrane embedded with three inkjet-printed electrochemical amperometric oxygen sensors to simultaneously monitor the oxygen level at the inflow (periportal-like), at the middle, and at the outflow (perivenous-like) of the hepatic culture chamber and to recapitulate liver zonation.

Regarding gut-on-chip systems, the establishment and maintenance of an oxygen gradient is of paramount importance to simultaneously allow culture of intestinal epithelial cells and microbiome in aerobic and anaerobic conditions, respectively. In fact, many bacteria fail to grow at oxygen concentrations greater than 0.5% that, on the contrary, are needed for intestinal cells maintenance [73,74]. To mimic this condition, Shah et al. [75] cultured Caco-2 cells and anaerobes in a modular microfluidic device (HuMiX, Figure 1b) composed by three microchambers: one for medium perfusion, one for human epithelial cell culture, and the last for microbial culture. A microporous permeable membrane separates the perfusion and epithelial microchambers, whereas a nanoporous membrane divides the epithelial and microbial microchambers. Optical oxygen sensors (0.03% O_2_ sensitivity, 5 mm diameter) were affixed to both upper and lower enclosures, 20 mm adjacent to the inlets and outlets of the perfusion and microbial microchambers. Simultaneous perfusion of oxic (21% dissolved O_2_) and anoxic (0.1% O_2_) media through the perfusion microchamber and the microbial microchamber, respectively, allowed the establishment and maintenance of an oxygen gradient representative of the in vivo situation. Optical oxygen sensors for the real-time noninvasive and noncytotoxic monitoring of the dissolved oxygen concentrations in a gut-on-chip device were also adopted by Jalili-Firoozinezhad et al [26]. In particular, they developed a multilayered microfluidic device comprising upper and lower cell culture microchannels with a microfabricated porous elastic membrane sandwiched in-between. Six sensor spots containing oxygen-quenched fluorescent particles were embedded at the inlet, middle, and outlet of both the top epithelial channel and bottom endothelial channel for monitoring oxygen tension. As the device is composed of highly gas-permeable PDMS, the sensors respond rapidly (<30 s) to changes in oxygen concentrations. To simultaneously provide an adequate oxygen level to maintain human cells and an anaerobic microenvironment suitable for culturing complex human microbiota, while establishing a functional host−microbiome interface, they flushed the upper chamber continuously with humidified 5% CO_2_ in nitrogen (N_2_) gas. This setup enabled the generation of physiologically relevant anaerobic conditions maintaining low oxygen levels (<0.5%) within the lumen of the upper chamber, while the epithelium was sustained via the diffusion of oxygen through the permeable PDMS membrane from the oxygenated medium flowing through the lower endothelial channel.

Recreating anaerobic culture environments in OoC systems can also be important in disease modeling. For example, Mauleon et al. [76] developed a microfluidic oxygenation device enabling to quickly and precisely adjust the spatial oxygenation conditions of subregions of mouse brain slices in order to mimic stroke events. In particular, they adopted ruthenium-based optical oxygen sensors to monitor O_2_ level in brain slices upon induction of a hypoxic insult. In fact, a hypoxic stimulus as short as 5 min can produce lasting damages to neuronal cells [77]. They were able to create a hypoxic environment (9% O_2_) throughout the entire slice in less than 4 min and with only an 8% difference from the bottom to the top of the slice (350 µm thickness). Similarly, Sticker et al. [78] generated a model of the blood–brain barrier (BBB) under hypoxia, to mimic acute ischemic stroke conditions. In their study, a microfluidic device based on thiol-ene-epoxy chemistry was developed as this material exhibits intrinsic oxygen scavenging properties to tune oxygen concentrations [79]. They adopted two complementary oxygen-sensing strategies: 5 µm palladium-based optical oxygen-sensitive microparticles attached to the surface of the device and an electrochemical oxygen-sensing method to directly detect dissolved oxygen levels by oxidation at the electrode surface. Both sensor systems were used to assess the ability of the material to tune oxygen level in the device by modifying geometric features and fabrication parameters of the thiol-ene-epoxy biochips (Figure 1c). In particular, oxygen scavenging rates could be readily adjusted over applied curing temperatures during biochip fabrication to remove oxygen from the device without the need for external incubation chambers or soluble chemical oxygen scavengers.

In summary, detecting the concentration of dissolved oxygen in cell culture medium is crucial for monitoring cell culture conditions in a variety of OoC applications as alterations or strong variations in organ-specific culture conditions may lead to unintended cell stress and damage [80].

### 2.2. Glucose and Lactate

Glucose is the primary energy source of cells, and it is metabolized into pyruvate or lactate under aerobic or anaerobic conditions, respectively. In particular, an increase in lactate concentrations is considered as an indication of cellular stress and disfunction. Thus, monitoring the concentration of glucose and lactate is important not only to gauge the reliability of the OoC setups, but also to provide insights about cellular health and function, cell viability upon toxicity [69,81], cancer cell progression, molecular basis of brain functions [82], cell metabolism [59], and dynamics of mitochondrial dysfunction (i.e., from oxidative phosphorylation to anaerobic glycolysis) [69]. 

In recent years, various types of glucose and lactate sensors have been developed for integration into OoC platforms [59,69,82]. Electrochemical biosensors dominate the field of enzyme-based biosensors. In these biosensors, specific enzymes (i.e., glucose or lactate oxidase) are incorporated as biorecognition elements and immobilized on a membrane (e.g., poly(2-hydroxyethyl methacrylate)-based (pHEMA)), directly placed on the sensing electrodes (usually platinum electrodes). Once such immobilized enzymes catalyze the corresponding analyte (i.e., glucose or lactate), hydrogen peroxide (H_2_O_2_) is produced and measured via voltammetric or amperometric methods, providing a direct correlation with concentration of the analyte of interest [83]. Compared to optical measurements, such electrochemical sensing modalities are preferred due to their ease of integration in microfluidic devices but require frequent recalibration and are often limited by sensor’s lifetime. To overcome the latter limitation, Misun et al. designed a hybrid microfluidic platform with interchangeable sensors. They developed a hanging drop PDMS device with removable sensor modules for the formation, cultivation, and simultaneous in situ monitoring of glucose and lactate of 3D colon microtissues, obtaining an outstanding sensitivity of detection upon applying different culture conditions [60].

**Figure 2:**
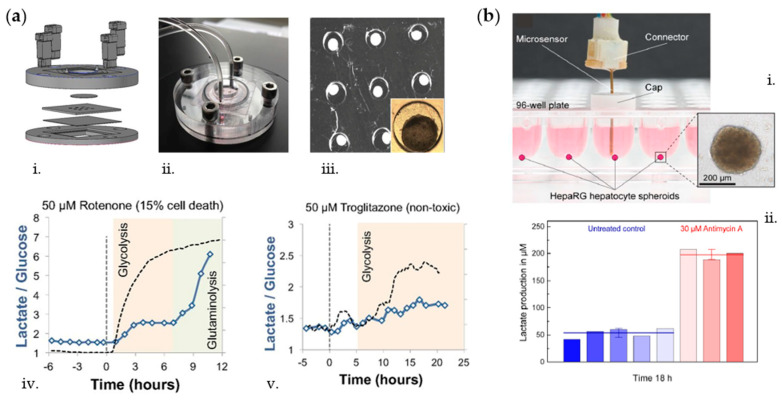
(**a**) Liver bioreactor components: bottom polymethyl methacrylate (PMMA) housing, cover glass, laser-cut PDMS microwells, glass window, and top PMMA cover (i); photo of assembled bioreactor (ii); composite tile scan image of HepG2/C3A organoid after overnight incubation (iii); changes in lactate over glucose ratio following exposure to rotenone (dotted line, iv); changes in lactate over glucose ratio following exposure to troglitazone (dotted line, v). Adapted from Ref. [69] with permission from PNAS. (**b**) Microsensor device for lactate monitoring placed in the 96-well cell culture plate (i) and lactate measurement of HepaRG spheroids exposed to 30 μM Antimycin A over 18 h (ii). Adapted from Ref. [81] with permission from Elsevier.

Electrochemical biosensors have also been exploited for monitoring toxicity caused by drugs. In that regard, Bavli et al. [69] developed a perfusion polymethyl methacrylate (PMMA) bioreactor (Figure 2a) integrating disposable PDMS microwell inserts for observing changes in hepatic spheroids upon exposure to mitochondrial inhibitor rotenone and antidiabetic drug troglitazone. By means of an off-chip electrochemical sensor unit, they achieved real-time glucose and lactate measurements showing increased lactate over glucose ratios after drug administration. Specifically, they demonstrated a strong shift toward glycolysis without impairing cell viability at low troglitazone concentrations (50 µM), below the threshold of clinical toxicity. In another work, Weltin et al. [81] developed electrochemical glucose and lactate sensors for continuous in situ monitoring of liver and brain cells culture conditions upon compounds dosage. In particular, they adopted the drug bosentan, clinically used to treat hypertension, to show a dose-dependent decrease in lactate production by 3D HepaRG spheroids, whereas antimycin A was used as model drug increasing hepatocytes lactate production (Figure 2b). Moreover, they fabricated a borosilicate glass chip for the culture of 2D T98G brain cancer cells and adopted the drug cytochalasin B to decrease cellular lactate production upon constant medium perfusion [59].

### 2.3. Cytokines and Other Metabolites

Lately, along with enzyme-based electrochemical biosensors, considerable efforts have been invested into the integration of electrochemical immunosensors within OoC platforms [84]. Several methods, such as voltammetry, amperometry, and impedimetric measurements have been used for developing electrochemical immunoassays with the goal of maximizing signal and sensor sensitivity [85]. At-line detection of cell-secreted molecules is of paramount importance for observing the changes in cellular/tissue metabolic activity. Cytokines, for instance, regulate different cell functions during immune responses such as proliferation, migration, or activation of cells and were shown to play critical roles in cancer progression as well as cell–cell signaling [86,87]. The most widely used in situ technique for cytokines and small proteins on-chip detection is based on immunoassay such as in antigen–antibody assays [61,88,89,90], aptamer-based assays [91,92,93], and bead-based assays [94,95].

Regarding the integration of antibody-based sensors into OoC platforms, many functionalization strategies have been developed to ensure efficient and reproducible antibody immobilization on the sensor electrode to increase sensitivity of detection [84]. For instance, Ortega et al. [61] developed a PDMS-based muscle-on-a-chip platform for the local monitoring of inflammatory cytokines interleukin-6 (IL-6) and tumor necrosis factor-alpha (TNF-α) produced by murine skeletal myoblasts. Their multiplexed electrochemical sensing system was placed at the outlet of the culture chip, and it contained antibody-functionalized amperometric sensors with a sensitivity in the nanogram per milliliter range. By electrically and biologically (i.e., lipopolysaccharides (LPS) administration) stimulating the cellular construct, they showed that secretion of these cytokines by skeletal muscle is regulated by exercise stress. Considering antibody-based assays, Shin et al. developed a microfluidic bioreactor for the culture of human liver organoids connected to a downstream impedance-based sensor chip for the electrochemical monitoring of important liver biomarkers such as albumin and glutathione-S-transferase-alpha (GST-α) [89].

**Figure 3:**
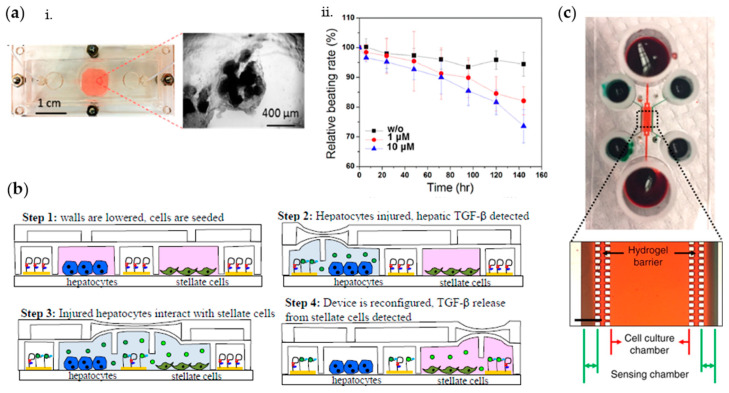
(**a**) Photograph of the microfluidic bioreactor and the cardiac spheroids (i); measured normalized beating rates of cardiomyocytes over time after exposure to different concentrations (0, 1, and 10 μM) of doxorubicin (ii). Adapted from Ref. [91] with permission from ACS Publications. (**b**) Aptamer-based two-layer device to monitor the communication between hepatocytes and stellate cells into three stages. Step 1: valves are closed to allow for cell seeding; Step 2: 100 mM EtOH in media infused into hepatocyte chamber to injure cells; Step 3: barrier raised to allow injured hepatocytes to communicate with quiescent stellate cells; Step 4: middle barrier lowered again to sequester stellate cells from injured hepatocytes and monitor TGF-β1 production from stellate cells. Adapted from Reference [92] with permission from The Royal Society of Chemistry. (**c**) Microfluidic system for the cultivation of hepatocytes and for the detection of secreted growth factors; photograph and microscopic image of the device; red and green food dyes were infused into the cell culture chamber and sensing chambers, respectively. Scale bar = 500 μm. Adapted from Reference [94] with permission from Springer Nature.

As being highly sensitive, selective, and thermochemically stable, aptamers have lately been preferred to antibodies for detection of proteins and cytokines. In this regard, Shin et al. [91] developed an aptamer-based electrochemical biosensing platform fluidically connected to a bioreactor culture chamber for monitoring damage to cardiac organoids. In particular, they functionalized the electrodes with aptamers specific to creatine kinase-MB biomarker whose concentration increased in a dose- and time-dependent manner when doxorubicin was administered in the system (Figure 3a). Moreover, Liu et al. quantified the inflammatory cytokines interferon gamma (IFN-γ) and TNF-α produced by activated T-cells cultured in a microfluidic device via anthraquinone and methylene blue redox reporters labelled aptamers on gold electrodes [93]. Appearance of the target cytokine at the electrode surface caused specific redox peak to shift downward in a concentration-dependent manner. Similarly, Zhou et al. [92] developed a microfluidic device integrated with aptamer-based sensors for continuous monitoring of transforming growth factor-beta (TGF-β) signaling in hepatocytes and stellate cells coculture upon alcohol injury (i.e., 100 mM ethanol for 48 h) (Figure 3b). The designed platform enabled the monitoring of paracrine crosstalk between two cell types communicating trough TGF-β and pointed out the importance of hepatocytes as early inducers of liver injury.

By combining magnetic microbead-based methods for analyte capture with fluidically connected electrochemical sensor units, Riahi et al. were able to measure the transferrin and albumin secretions of hepatic spheroids cultured in a bioreactor to monitor acetaminophen-induced toxicity over liver cells for up to 5 days [95]. In a more recent work, Son et al. [94] adopted fluorescent microbead-based sensors to monitor secretion levels of hepatocyte growth factor and TGF-β1 over primary hepatocytes for 7 days. The developed microfluidic system enables local cytokine monitoring from cells that are separated from sensing channels via a permeable hydrogel barrier (Figure 3c). As compared to previously reported electrochemical sensing approaches, the microbeads do not suffer from saturation problems as they can be replaced with new ones after completing one cycle of detection; therefore, the bead-based approach can be usable over many measurements.

## 3. Biosensors for Measuring OoC’ Endothelial and Epithelial Barrier-Related Features

Cell monolayers, including endothelial cells lining the vasculature and the blood–brain barrier (BBB), and epithelial cells lining the lung airways and the gut, play a crucial role as selectively permeable cellular barriers regulating the transport of biomolecules to underlying tissues and structures [96]. In fact, both endothelial and epithelial cells are connected to each other through tight junctions that control the transport processes, regulate paracrine communication between cells, and contribute to maintain organ homeostasis [97]. Although barrier integrity is thus critical for the functionality of the aforementioned tissues, it may also represent an obstacle when administrating drugs targeting organs protected by one of these barriers. Hence, the assessment of barrier function and integrity is of paramount importance when culturing endothelial or epithelial lining layers. Transepithelial/transendothelial electrical resistance (TEER) is the measurement of electrical resistance across a cellular monolayer, and it is a widely accepted technique to noninvasively quantify barrier integrity and tightness during the various stages of cells growth and differentiation. The integration of TEER measurements in OoC models represents a promising strategy for assessing drug toxicity and predicting barrier permeability to specific drugs [98].

**Figure 4:**
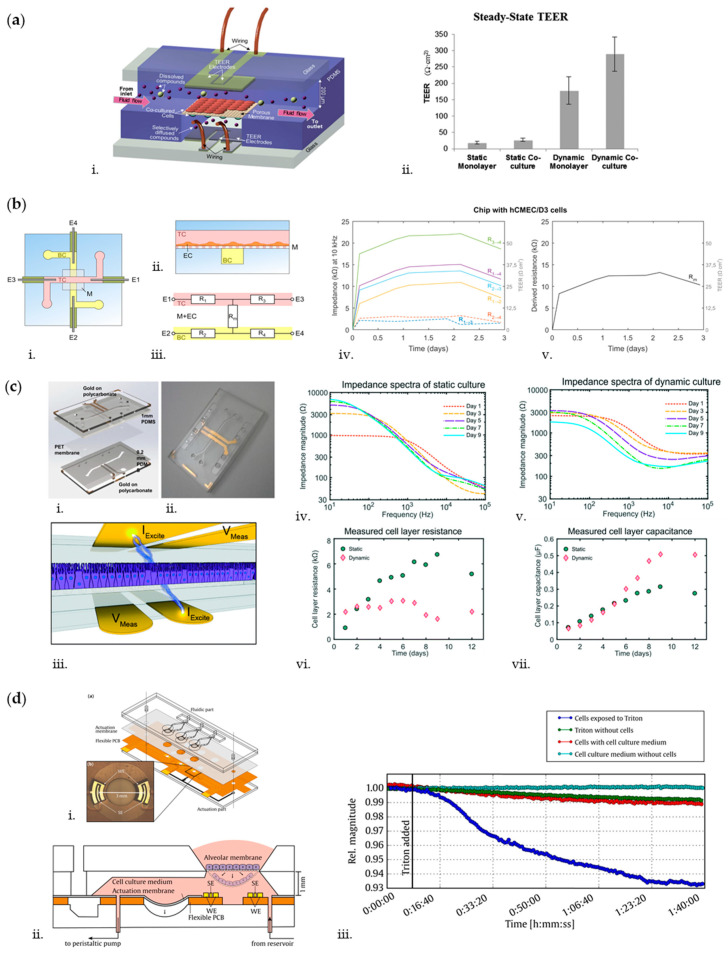
(**a**) 3D illustration of the microfluidic blood–brain barrier (BBB) (i), composed of two perpendicular flow channels (lumenal on top and ablumenal at the bottom), separated at the channel junction by a porous membrane. Endothelial cells and astrocytes are, respectively, cultured on the lumenal and ablumenal sides of the membrane. Steady-state transepithelial/transendothelial electrical resistance (TEER) levels (ii) show that dynamic cultures (µBBB) reached significantly higher TEER levels than static cultures (Transwell controls). For both systems, cocultures of endothelial cells and astrocytes developed higher TEER levels than endothelial monolayers alone. Adapted from Ref. [108] with permission from The Royal Society of Chemistry. (**b**) Schematic top view (i) and side view (ii) of the chip to perform impedance spectroscopy measurements, showing top channel (TC), membrane (M), bottom channel (BC), platinum wire electrodes (E1, E2, E3, and E4), and endothelial cells (EC). The simplified equivalent circuit of the chip (iii) show resistors representing the top channel (R_1_ and R_3_), resistors representing the bottom channel (R_2_ and R_4_), and resistor R_m_ representing the membrane and EC barrier. Impedance and TEER measurements at 10 kHz for each electrode pair in a chip with hCMEC/D3 cells (iv) show a large variation between the electrode pairs. By calculating R_m_ (v) the resistance of the membrane and cells is isolated. Adapted from Ref. [115] with permission from Elsevier. (**c**) Exploded CAD (Computer-Aided Design) model (i) and photograph (ii) of the TEER-chip developed by Henry et al. Gold electrodes are patterned onto polycarbonate substrates. Laser cut polydimethylsiloxane (PDMS) layers and PET membrane are assembled using silane-based surface modification to irreversibly bond together. The schematic view of the chip and 4-point impedance measurement (iii). A small current of varying frequency is applied between two electrodes (I_excite_) located on each side of the cell monolayer, and the drop in potential between the second set of electrodes is measured (V_meas_). Impedance spectra obtained during the 12-days culture period of Caco-2 epithelial cells show the development of an intestinal epithelial barrier cultured under static (iv) or dynamic flow (v) conditions from days 1–9. The epithelial resistance (vi) of the statically cultured cell layer increased in time up to a plateau, while the resistance of the cell layer cultured under flow decreased after day 7. The capacitance (vii) of the static epithelium reached a plateau after day 8, while the capacitance of the epithelium cultured under dynamic flow conditions continued to increase. Adapted from Ref. [118] and Ref. [119] with permission from The Royal Society of Chemistry. (**d**) Design of the microimpedance tomography (MITO) and integration in the lung-on-chip (i). The MITO consists of a flexible printed circuit board (PCB) bonded between the actuation part and the actuation membrane of the lung-on-chip. It comprises three sensing regions, each consisting of a tetrapolar arrangement of two pairs of electrodes. The sensing electrodes (SE) of each pair are located in the center of the working electrodes (WE) to avoid zones of negative sensitivity. The cross-sectional view of the system (ii) shows a culture chamber containing the alveolar membrane, the actuation membrane, and the SE and WE of the MITO located 1 mm below the cell culture membrane. Microchannels connected to a reservoir enable the injection of solutions into the basal compartment. A time-lapse of the relative impedance magnitude at a frequency of 1 kHz (iii) reports changes in relative impedance of the bronchial epithelial monolayer after exposure to the permeabilization solution of 0.5% Triton X-100. Adapted from Ref. [121] with permission from Elsevier.

The BBB is a highly selective barrier between the peripheral circulatory system and the central nervous system (CNS), whose function depends on close interactions between adjacent brain microvascular endothelial cells [99,100,101]. It plays a crucial role in maintaining brain homeostasis and its dysfunctions have been associated with various neurological disorders [102,103,104,105]. Thus, in the last decade, OoC platforms have been developed to recapitulate the BBB microenvironment and to monitor barrier integrity through TEER measurements, aiming at studying the mechanisms of BBB and evaluating drugs targeting the CNS [106,107]. One of the first microfluidic BBB that closely mimicked the dynamic cerebrovascular environment was developed by Booth and Kim [108]. They presented a multilayered microfluidic device, housing two perpendicular channels separated at the channel junction by a porous polycarbonate membrane (Figure 4a). The two channels, seeded, respectively, with endothelial cells (b.End3 cell line) and astrocytes (C8D1A cell line), modeled the lumenal and ablumenal sides of the neurovascular unit. A peristaltic cartridge pump was used to introduce dynamic flow in the top channel (2.6 µL/min) inducing a shear stress of 1.6 dyn/cm^2^ on the endothelial cells, to recapitulate the in vivo mechanotransductive effects, which play a key role in tight junction formation [109]. To measure TEER as an indicator of cell confluence and tight junction integrity, each channel hosts a set of two AgCl thin-film electrode pairs fabricated through sputter deposition, forming a four-point sensing structure. The electrode wires are connected to an EVOM2 epithelial volt/ohm meter (World Precision Instruments, Sarasota, FL, USA) that passes a constant 10 µA AC square wave at a frequency of 12.5 Hz while measuring the electrical resistance of the cellular layers. TEER was obtained as:

$$\mathrm{TEER}=(R_{C}-R_{b})A\;\;\;\left[{\Omega *\mathrm{cm}}^{2}\right]$$

where $R_{C}$ is the total resistance at the considered time point, $R_{b}$ is the blank resistance of the semipermeable membrane only, and A is the area of membrane. Notably, by day 3–4 after seeding, a TEER exciding 250 Ω cm^2^ was measured. This value indicated the formation of a functional endothelial layer, being considerably higher as compared to Transwell controls (25 Ω cm^2^) and acceptable for BBB in vitro models [110]. As further proof of the robustness of their solution, they observed the TEER values in real-time after exposing the cells to histamine, showing an expected transient drop and a very rapid recovery (6–15 min) to the original TEER levels. This result proved one of the main advantages of this system, i.e., the ability to perform real-time TEER measurements in a noninvasive way. 

A similar solution to assess barrier permeability was reported by other groups [37,111,112]. However, a more accurate representation of TEER can be obtained using impedance spectroscopy, that consists in applying a small amplitude AC excitation signal with a frequency sweep and measuring the amplitude and phase response of the resulting current. By fitting the experimental spectrum data to equivalent circuit models using nonlinear least-squares fitting techniques, it is possible to obtain information both about TEER and the capacitance of the cell layer [98]. This solution has been widely adopted within OoC platforms in the last decade [113,114,115]. For instance, van der Helm et al. [115,116] proposed a method to perform impedance spectroscopy measurements without the need to integrate electrodes in proximity of the cellular barrier, generally hampering cells visual inspection (Figure 4b). The microfluidic platform consists of two PDMS layers, forming two perpendicular channels separated by a polycarbonate membrane. A pair of platinum wire electrodes is inserted in each microchannel, so that a sufficiently large surface area of the electrodes is exposed to culture medium and an AC signal scanning from 200 Hz to 1 MHz is used for impedance measurements. From the measured impedance spectra, the resistances between the electrodes are then determined from the resistive plateau at 10 kHz. In this configuration, however, the TEER value cannot be measured directly, but needs to be computed by normalizing the resistance of the cellular barrier and of the membrane $R_{m}\;$(obtained by indirect Gaussian elimination) for the culture area and by subtracting the background TEER. As proof-of-concept, hCMEC/D3 cerebral endothelial cells (provided by P.-O. Couraud, INSERM, Paris, France) were cultured on top of the membrane and TEER was measured for several days, obtaining an average of 22 Ω cm^2^ by day 3, a value close to other TEER values reported for this cell type, but lower than the TEER value obtained in the previously described BBB model developed by Booth and Kim’s, probably due to the absence of astrocytes and of fluid flow. However, van der Helm et al. proved that TEER can be monitored while keeping the electrodes far from the culture area, thus reducing variation due to parameters of nonbiological origin, such as temperature and medium composition. This method is potentially applicable to any OoC presenting two channels and a membrane in between, but more efforts should be done to make this technique compatible with OoCs standard setup (e.g., integrating fluid flow).

The aforementioned techniques have been widely reported in literature to measure TEER also inside OoCs recapitulating the intestinal epithelium. The epithelial layer of the intestinal mucosa is considered to be the limiting hurdle for gastrointestinal drug permeation and is therefore a component of high interest for studying in vitro drugs targeting the gut [98]. In this context, measurement of TEER is still considered as a quick and noninvasive assay to evaluate the formation of tight junctions between neighboring epithelial cells. A pivotal intestinal microphysiological model integrating TEER was reported by Kim et al. [117], which developed a human “gut-on-a-chip” undergoing peristalsis, experiencing fluid flow, and supporting growth of microbial flora. The device consists of a central cell culture channel, which in turn presents an upper and a lower channel separated by a porous membrane, and two lateral vacuum chambers. Within this setup, fluid flow was imposed at a constant flow rate through the central channels (30 µL/h) to produce a low shear stress (0.02 dyne/cm^2^) on the Caco-2 epithelial cells (Harvard Digestive Disease Center, Boston, Massachusetts) seeded on the membrane, while mechanical cyclic strain (10%; 0.15 Hz) was exerted by applying suction to the vacuum chambers. The TEER of the epithelial monolayer was measured using Ag/AgCl electrode wires connected to a volt/ohm meter, obtaining by day 6 TEER values of up to 3000–4000 Ω cm^2^ in the microfluidic system, whereas control Transwell cultures (static) exhibited TEER values between 700 and 800 Ω cm^2^. Likewise, for BBB models, impedance spectroscopy measurements would however be preferable to obtain a more accurate representation of TEER in the intestine. To this aim, Henry et al. [118] designed a microfluidic device consisting of two PDMS laser-cut layers, separated by a porous PDMS membrane, interposed between two polycarbonate substrates patterned with integrated semi-transparent gold electrodes (Figure 4c). Caco-2 epithelial cells were cultured on top of the porous membrane as a flat monolayer when cultured statically, or in villus structures when cultured under flow by connecting the inlets of both channels to a syringe pump and imposing a medium flow rate of 30 µL/h [119]. Electrochemical impedance spectroscopy in galvanostatic mode using four-point measurements was used to evaluate TEER. Specifically, two excitation electrodes were used to excite the cells with 10 µA current in AC ranging from 10 Hz to 100 kHz, while two measuring electrodes were used to record the difference of potential. The impedance curves were then fitted to an equivalent electric model to extract TEER and cell capacitance values. With this setup, it was showed that during static culture, the monolayer resistance increased in time reaching values of 7000 Ω by day 9, whereas during dynamic culture, the resistance averaged 3000 Ω at day 6 and then it started decreasing, coinciding with formation of 3D villi. It should be noted that the TEER measures are here presented as Ω, and not as Ω*cm^2^. According to the authors, the device measures resistance of the cell layer area directly in between the electrodes, but it is also likely influenced by regions of the culture area outside the edges of the electrodes. Thus, they proposed not to normalize the resistance by the surface area of the cultured tissue being analyzed. However, the major advantage of this device is the robustness of the electrode setup, which decreases the variability among devices with the same design and increases the measurement reliability. Furthermore, this design can be ideally used to recapitulate any type of endothelial or epithelial lining layer and to monitor its integrity in real time. In fact, it was also used to reproduce and monitor the development of a lung epithelium by culturing primary human airway epithelial cells (hAECs) in the upper channel at an air liquid–interface (ALI) and feeding them by flowing growth medium through the lower channel.

Overall, the study of barrier integrity through TEER measurements exhibits some disadvantages. First, the electrodes may limit the field of view and measurements are sensitive to electrode properties and positioning, to electric field uniformity, and to the electrical circuit model used to interpret the data [120]. Furthermore, a limitation is represented by the need of completely submerging the model in culture medium to ensure electrical connection, that may represent an issue when culturing epithelial cells that require air–liquid interfaces. To solve this problem, Mermoud et al. [121] developed a microimpedance tomography (MITO) system integrated in a lung-on-chip to monitor the electrochemical changes occurring in the lung alveolar barrier (Figure 4d). Each device consists of three culture chambers, containing a suspended porous PDMS membrane, on which human bronchial epithelial cells (16HBE14o-) (provided by Gruenert, University of California, San Francisco, California) are cultured and that is cyclically deflected to replicate the respiratory movements. The deflection is indirectly generated by applying vacuum to an actuation membrane located at the bottom of the cell culture chambers. The MITO consists of a flexible printed circuit board (PCB) (Margit Medinger Electronic, Germany) located under the actuation membrane, with three impedimetric sensing regions (one in correspondence of each chamber). Each region presents a planar tetrapolar arrangement of two pairs of electrodes (two working and two sensing electrodes), so that there is no need for apical electrodes. The electrodes are located at 1 mm from the membrane. Impedance measurements were performed by injecting 15 µA AC current scanning from 100 Hz to 100 kHz. As proof-of-concept, the cell monolayer was permeabilized with Triton X-100 and a decrease of up to 10% in impedance magnitude was detected, showing that the system can successfully monitor in real-time resistivity changes occurring on the lung alveolar epithelial barrier.

Another major disadvantage of TEER measurements is the difficulty in interpreting and comparing TEER values between different devices, because of the intrinsic variability due to the measurement setup. Thus, tracer-based permeability assays remain still complementary to TEER measurements to get a robust characterization of barrier function [122]. However, permeability assays usually rely on intensive manual sampling and off-chip measurements, and the use of tracer compounds may interfere with the transport process under study and affect barrier integrity [96,98]. To overcome these issues, Wong and Simmons [96] reported an innovative on-chip electrochemical microfluidic permeability assay to assess barrier function. The microdevice is composed of four layers, i.e., two PDMS layers hosting two microchannels separated by a PET (polyethylene terephthalate) porous membrane and a glass substrate with patterned gold electrodes. The assay is performed by injecting a concentrated electroactive tracer solution (hexaamineruthenium chloride, RuHex) into the upper channel and electrochemically monitoring the accumulation of the tracer in the lower channel over time, using an electroanalytical technique called square wave voltammetry (SWV). SWV scans are continuously performed until the peak SWV current reaches a maximum value, indicating that diffusive transport of the tracer has ceased. SWV peak currents are then normalized by this maximum peak current and the resulting electroactive tracer transport kinetics are obtained by plotting the normalized peak current versus time. From these values, the diffusive permeability of the tracer molecule across the cell monolayer can be computed. As proof-of-concept, primary porcine aortic endothelial cells (PAECs) (provided by Lowell Langille, University of Toronto, Toronto, Canada) were seeded on top of the porous membrane and endothelial permeability was assessed under static and perfused conditions (0.01 dyn/cm^2^ for 72 h). The static results showed a slower accumulation of the tracer (measured permeability of 3.46 ± 1.05 × 10^−5^ cm s^−1^) as compared to a cell-free porous membrane (measured permeability of 8.51 ± 0.91 × 10^−5^ cm s^−1^) and an increase in transport dynamics if the cell layer was treated with EGTA, a well-known chelator (measured permeability of 5.38 ± 1.09 × 10^−5^ cm s^−1^). A good accordance was shown between these results and control measurements, obtained using a fluorescent tracer in a Transwell assay. The permeability value of PAECs cultured under perfusion (2.80 ± 0.15 × 10^−5^ cm s^−1^) was lower than the static one, suggesting that a constant nutrient supply and waste removal is helpful to improve barrier function. In conclusion, this permeability assay is a method to evaluate barrier function and integrity, that combines the robustness of tracer-based permeability assay with the benefits of on-chip integration. 

## 4. Biosensors for Measuring OoC’ Electrical Activity

The main biological model with measurable electrical activity that can be recapitulated on-chip is the nervous system. Neurons and their extensions grow in vivo in a densely packed, highly organized, and 3D environment, and they typically extend over considerable distances and through different microenvironments [123,124]. To overcome the limits of conventional neuronal cultures, advanced in vitro models are required to recapitulate the in vivo biological complexity while allowing for (i) control of the extracellular environment with adequate spatial and temporal resolution and (ii) high-resolution analysis of subcellular signals [125]. In particular, the advent of compartmentalized microfluidic devices has revolutionized the field of neurobiology. Over the past two decades, various microfluidic platforms have been developed to investigate axon growth, synapses formation, as well as neural injury and neurodegenerative disorders [38,39,126,127,128,129,130,131,132].

Once a neural circuit is implemented on chip, the possibility to record the electrical activity of the neuronal populations in real time is an essential requirement to acquire a basic understanding of the network functionality. To this aim, several studies have successfully demonstrated the integration of commercial or custom-made multielectrode arrays (MEAs) within microfluidic chips [53,133,134,135,136,137,138]. In details, MEAs are composed of a set of microelectrodes typically arranged in a matrix configuration, that enable to record extracellular activity of neuronal populations from multiple sites simultaneously in a noninvasive way [139,140,141]. The combination of MEAs with compartmentalized microfluidic platforms allows to study electrical activity of neuronal populations with high spatial resolution, as electrode interfaces can be matched with specific cell sites (e.g., soma and axons) [142,143,144].

**Figure 5:**
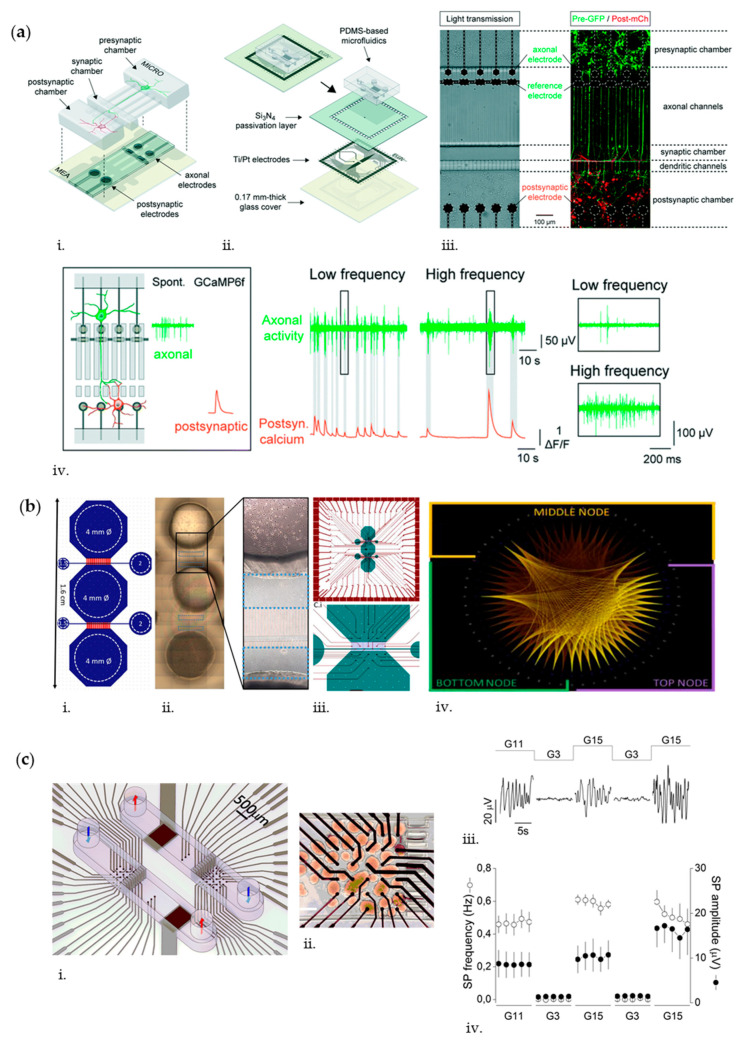
(**a**) Design of the microMEA platform: scheme showing the microfluidic and microelectrode components of the platform (i) and exploded view showing the different lithography layers for the fabrication of the multielectrode array (MEA) (ii). The light transmission and fluorescence images of the device (iii) show the positions of axonal, reference, and postsynaptic electrodes, and a reconstructed cortico-cortical network with GFP (Green Fluorescent Protein)-expressing axons and mCherry-expressing postsynaptic dendrites at DIV 10. Monitoring postsynaptic calcium dynamics via high-resolution microscopy in response to presynaptic electrical activity of a single projecting axon (iv) show that weak postsynaptic calcium signals are related to projecting axons with low-frequency activity, whereas larger calcium signals are related to projecting axons with high-frequency activity. Insets show magnification of boxed areas. Adapted from Ref. [136] with permission from The Royal Society of Chemistry. (**b**) Design of the three-nodal microfluidic chip (i) and tiled brightfield images of all cell compartments (ii), with a magnification showing a zone between two nodes. Design of MEA layout aligned with the 3-nodal chip design and magnified image of internodal area (iii). The functional map of the connectivity (correlation of concurrent spikes) between electrodes in the 3 nodes at DIV 18 show high degree of internodal connectivity (iv, green: top node, purple: bottom node, and orange: middle node). Adapted from Ref. [53] with permission from Elsevier. (**c**) Scheme of microfluidic MEAs for the analysis of pancreatic islets (i), showing two symmetric chambers. Each chamber has two compartments separated by microchannels, 1 with 26 recording electrodes and 1 with 3 control electrodes and 1 reference electrode. Microscopic image of intact islets 4 days after seeding in the microfluidic MEA chamber shows good electrode coverage (ii, scale bar 200 µm). Representative recording of islets’ slow potentials (iii) obtained 2 h after seeding on microfluidics MEAs under different glucose concentrations (RPMI medium (Roswell Park Memorial Institute) containing 11 mM glucose or buffer containing either 3 or 15 mM glucose) show islets glucose-dependent electrical activity. Frequency and amplitude analysis of slow potentials are also reported (iv), considering the last 5 min of each condition (lasting 10 min) and providing mean data for each minute. Adapted from Ref. [153] with permission from Elsevier.

For instance, Moutaux et al. developed an on-chip platform that combines a compartmentalized microfluidic device with a dedicated MEA to selectively record electrical activity in presynaptic axonal projections and postsynaptic neuronal targets, while simultaneously studying intracellular dynamics via high-resolution videomicroscopy (Figure 5a) [136]. The microfluidic platform consists of two opposite neuronal chambers connected via an intermediate synaptic chamber through microchannels of different lengths, i.e., 500 µm for presynaptic neurons and 75 µm for postsynaptic neurons [132]. The microfluidic chips were plasma bonded to MEA platforms, specifically fabricated to fit the layout of the microfluidics: in details, each MEA is composed of a glass substrate with a set of 59 Ti/Pt electrodes on top, being 30 (30 µm diameter) positioned in the upper part of the presynaptic microchannels and 29 (50 µm diameter) positioned in the postsynaptic cortical chamber, plus 1 line of grounded reference for both pre- and postcompartments. The electrodes were connected to a commercial amplifier (MEA-1060-Inv-BC amplifier, MultiChannel Systems, Reutlingen, Germany) via connecting pads, and impedance measurements are carried out using an impedance analyzer. To validate this setup, a corticocortical network was reconstituted from rat embryonic neuronal progenitors and spontaneous electrical activity in mature cultures (>DIV 12) recorded. Notably, measured activities were similar to the ones of pyramidal and inhibitory interneurons in vivo, i.e., neurons with phasic activities, showing episodes of low-spiking activity alternating with bursts of action potentials at high frequency, and neurons with tonic spiking activity at a lower frequency, respectively. As additional validation, they linked calcium dynamics of the postsynaptic neurons, recorded via high-resolution microscopy, with presynaptic axonal electrical activity and showed that weak postsynaptic calcium signals were related to projecting axons with low-frequency activity, whereas larger calcium signals could be detected in postsynaptic neurons connected to high-frequency axons. Overall, these results proved that the platform can be used to reconstruct functional and physiologically relevant neuronal network, to record neuronal activity and to study how axonal activity patterns influence the behavior of postsynaptic targets [136]. Similarly, van de Wijdeven et al. [53] developed a three-nodal microfluidic chip integrated with a custom-designed and in-house fabricated MEA to monitor electrical activity and intra-/ internodal connectivity of organized neuronal networks (Figure 5b). The microfluidic chip is characterized by three open cell compartments (i.e., the nodes), interconnected via microchannels, and two synaptic channels perpendicular to the microchannels. The customized MEA consists of 59 Pt/Ti recording electrodes and an internal reference electrode, deposited on a borosilicate glass substrate. The electrodes have a surface area of 2352 µm^2^ and are intended to obtain information from neuronal subpopulations within each node, as well as from the isolated neurites. The electrodes layout was designed to align with the microfluidic chip, with 10 electrodes located in the lateral nodes, 11 in the middle one, 5 underneath the microchannels in each internodal area, and 4 in each synaptic channel. 

The layout and contact points for data acquisition are compatible with a commercial recording system for impedance measurements (MEA-IT60, MultiChannel Systems, Reutlingen, Germany). Raw MEA data were processed off-line using NeuroExplorer (5th ed, Nex Technologies, Vienna, Austria) to detect the spikes and compute the mean spike rate, while a MATLAB (2018, MathWorks, Natick, Massachusetts) code was used to determine the functional connectivity of the neuronal network starting from these data. The recordings were also analyzed for bursting activity. As a demonstration, the authors seeded rat primary cortical astrocytes and neurons within each node and analyzed the extracellular action potentials data obtained from the MEA by DIV 18 burst activity and was synchronized with bursts propagating from one node to another through the microchannels, resulting in functional internodal connectivity. In addition, they could measure neuronal spike activity in response to selective neuromodulation of single nodes, as well as axotomy of neurites in the synaptic channel. All together, these results showed that the proposed system is a versatile platform, which can be exploited to study electrical activity and functional development of neuronal networks in physiological and pathological conditions.

Another biological model in which the integration of electrical sensors can be crucial is pancreas. According to the physiology of pancreatic islets, β-cells sense glucose, metabolize it to secrete insulin, and are nevertheless electrically excitable. In fact, during the multistep process of glucose metabolism, ATP is produced, which in turn controls the opening and closure of specific ion channels and triggers well-defined ion fluxes that generate an electrical current [145,146]. The electrical signals produced from pancreatic islets are of two types: short-lived high-frequency action potentials (AP) that reflect single cell depolarization and longer lived changes in field potentials, named “low frequency slow potentials” (SP) [147]. In particular, SPs consist of a summation of single cell activity and reflect intraislet coupling, which represents a major index of physiological quality in insulin secretion [148,149,150,151]. Moreover, SPs carry information about glucose concentration, with their frequency being directly correlated to the nutrient concentration: for instance, Pedraza et al. proved that, in murine islets, SPs are almost absent at low glucose (3 mM) levels, while their frequency increases for higher glucose (15 mM) stimulation [145]. Furthermore, Lebreton et al. found a positive correlation between SP frequencies and glucose concentration in murine islet cells, as well as in human islets [148]. Various works in literature developed the use of extracellular recordings using MEAs to capture the electrophysiological changes of pancreatic islets, taking advantage of the unrivalled temporal resolution of these recording systems [147,148,150,152]. On the other hand, there are limited works combining microfluidics and MEAs to study pancreas-on-chip models. For instance, Perrier et al. [153] combined electrophysiology, microfluidics, and microelectronics to develop a bioelectronic organ-based sensor for microfluidic real-time analysis of the demand in insulin with a microsecond latency (Figure 5c). The microfluidic platform consists of two antiparallel channels, that may be used to study simultaneously two biological samples. Each channel is divided into two chambers, one culture/recording chamber and one reference chamber. The two chambers are connected by 50 µm wide channels, which serve as barriers to block the islets and prevent them from migrating in the reference chamber. The sensing principle is based on microfluidic MEAs, composed of two symmetric chambers made of SU-8 epoxy, each divided in two compartments: one compartment with a grid of 26 recording platinum black electrodes and one compartment with 3 control planar electrodes and 1 reference ground electrode. Each MEA fits the layout of the microfluidic chips, so that, after plasma bonding, the recording electrodes are located in the culture/recording chambers, while the others are placed at the bottom of the reference chamber. Each assembled device is then connected to a microfluidic pump, to impose a perfusion of 5 µL/min. The complete setup permits real-time extracellular recordings of APs and SPs via an amplifier and the in-house built MULTIMED Field-Programmable Gate Array consisting of an acquisition and a signal analysis board. As validation, they seeded murine pancreatic islets in the culture chamber and examined their behavior 2 h after seeding upon stimulation with low (3 mM) and high (11 and 15 mM) levels of glucose. In agreement with literature data, no electrical activity was present at low glucose levels, while the response increased in terms of frequency and amplitude when the cells were stimulated with high levels of glucose, thus indicating an enhanced islet activity. Furthermore, the platform was able to detect a biphasic electrical activity, typical signature of human or murine islets in vivo, as well as in vitro. Overall, these results proved that physiological recordings of the islet electrical activity could be obtained within 2 h after seeding, a very short period as compared to several days required for classical MEAs.

## 5. Biosensors for Measuring OoC’ Mechanical Activity

**Figure 6:**
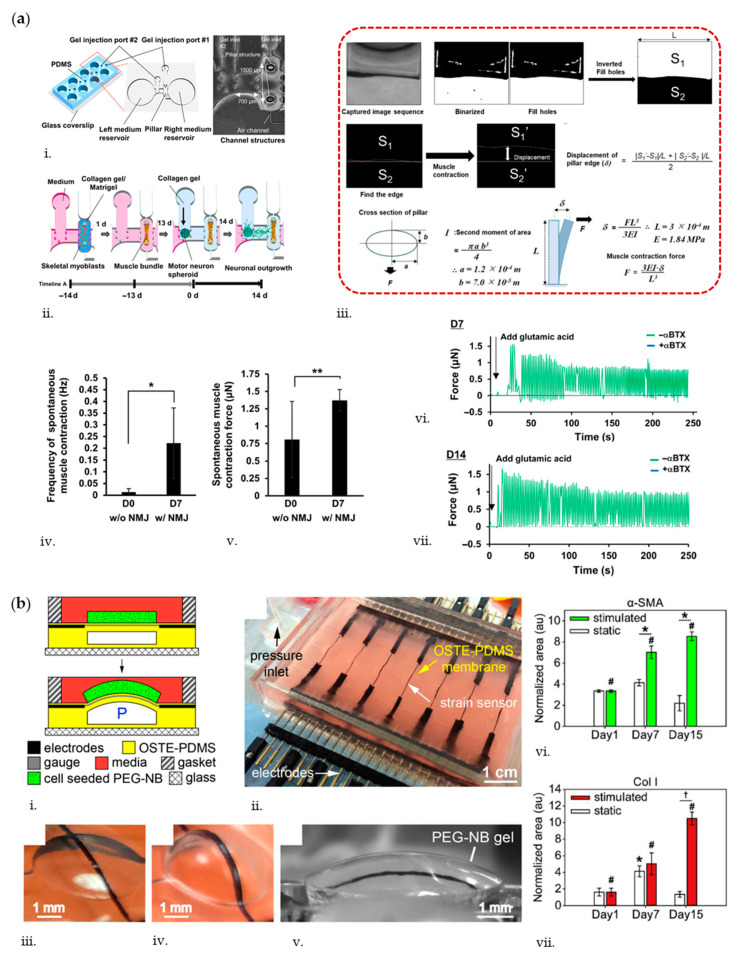
(**a**) Compartmentalized design and picture of a human motor unit on a chip microfluidic device. PDMS microchannels are used to form four identical sites on a single chip, each composed of a muscle fiber bundle attaching pillar structures and culture motor neuron (MN) spheroids. Each site has two medium reservoirs, two gel injection ports, and three compartments (i). Each device has three distinct culture regions: for MN spheroids, muscle tissues, and neurite elongation in the middle. The distance between two pillars is 1500 μm. Schematic of human induced pluripotent stem cells (iPS)-derived skeletal myoblasts seeding (ii): cells injected into the right compartment with the collagen/Matrigel mixture from gel injection port 1. Within 1 day, a skeletal muscle fiber bundle was formed on pillar structures. After 13 days of differentiation, an MN spheroid with collagen gel was injected into the left compartment from gel injection port 2. Neural outgrowth occurs by 14 days, resulting in the formation of a human motor unit along with neuromuscular junction (NMJ). In the dotted panel (iii), the process for automated detection of pillar displacement is reported, estimating muscle contraction. Estimation of spontaneous muscle contraction frequency (iv) and contraction force (v) on D0 (before NMJ formation) and D7 (after NMJ formation) shows a more frequent and stronger muscle contraction in presence of NMJ (n = 6, ** *p* < 0.05; * *p* < 0.01, Student’s *t* test, and one-way ANOVA. Error bars ± SD). Measurement of muscle contraction force in presence or absence of glutamic acid on D7 (vi) and D14 (vii) shows that muscle contraction is completely inhibited in presence of α-bungarotoxin (αBTX). Adapted from Ref. [30] with permission from American Association for the Advancement of Science. (**b**) Deformable membrane platform with integrated carbon nanotube (CNT) strain sensors for simultaneous 3D mechanical stimulation and measurement of cell-seeded hydrogels. (i) Device composition and operation; (ii) picture of a microdevice with integrated CNT sensors. Scale bar is 1 cm; example of a single deformable membrane unit with embedded CNT sensor at rest (iii), deformed (iv), and deformed with the bound polyethylene glycol (PEG)-norbornene (NB) gel (v); quantities of projected area normalized to nucleus were used to characterize cell responses for (vi) alpha-smooth muscle actin (α-SMA) (* *p* < 0.001, # *p* < 0.05) and (vii) Col I (* *p* < 0.05, # *p* < 0.05). Adapted from Ref. [55] with permission from Elsevier.

Monitoring tissue forces is relevant for OoC aiming at recapitulating dynamic deformations typical of the striatal muscular tissues. Striatal muscles in the human body can be categorized in skeletal and cardiac muscle tissues. Skeletal muscle is the largest organ in the human body and most of common musculoskeletal disorders lead to loss of functions, loss of regenerative properties, and significantly impaired strength and mobility [154]. From the anatomical point of view, skeletal muscle fibers contain sarcomeres that contract to generate forces that provide motion required in daily life. Engineered in vitro OoC systems recapitulating such physiological contraction properties have been recently developed. For instance, Agarwal et al. developed a muscle-on-a-chip device to generate uniaxially-aligned, densely packed 3D muscle tissue bundles to resemble native skeletal muscle [31]. Constructs composed of C2C12 mouse murine myoblast cells embedded in polyacrylamide (PAm) gel were confined around two anchoring PAm pillars and sandwiched between two top and bottom PAm layers used to quantify tissue generated strains and forces. Specifically, the deformation of top/bottom hydrogels due to tissue contractile stresses was quantified by tracking the displacement of fluorescent particles embedded within the gel. Beads movement was analyzed by particle image velocimetry (PIV) to obtain the passive tensile forces (mean 8.16 ± 3.41 μN, n = 7). Additionally, cardiotoxin (CTX)-induced changes of the engineered skeletal muscle tissue were evaluated by administering CTX from *Naja mossambica mossambica* (Sigma, C9759) at concentrations of 0.1–0.5 μM to the tissue under continuous flow (40 μL/h). Such CTX, commonly used in animal experiments to induce skeletal muscle injury, induced a decrease in passive tensions of exposed tissues in a dose-dependent manner. Another example to monitor skeletal muscle contraction was reported by Osaki et al. [30], who developed a compartmentalized device for the coculture of iPSC-derived skeletal myoblasts and motor neuron (MN) spheroids obtained from hESC-derived neural stem cells (NSC), recapitulating the neuromuscular junction (NMJ). The PDMS-based device consists of four identical culture sites, where each site has two medium reservoirs and three gel compartments delimited by pillars, hosting MN spheroids, neurites, and myoblasts, respectively. The last compartment presents capped pillar structures that serve as anchoring sites for muscle bundle formation. Muscle contraction force was estimated from optical measurements of the displacements of flexible pillars, automatically detected by analyzing captured videos using Python with an OpenCV package (Figure 6a). Results showed that before NMJ formation, the muscle contraction was less frequent (less than 0.05 Hz) and weaker (≈0.8 µN) than after NMJ formation (0.1–0.3 Hz, ≈1.4 µN). To test whether muscle contraction was triggered by MN activity, MNs were chemically stimulated through glutamate (0.1 mM), in presence or in absence of α-bungarotoxin (αBTX), an nAChR antagonist. By day 7, muscle contraction in absence of αBTX was observed and muscle force increased by day 14. On the other side, muscle contraction was completely inhibited in presence of αBTX, proving that muscle contraction was specifically triggered by nervous stimuli, while not by spontaneous muscle activity. These results showed that a physiological model of the motor unit with functional NMJs could be obtained. The same platform was used to reconstitute an amyotrophic lateral sclerosis (ALS) motor unit and to analyze muscle contraction forces in pathological conditions.

In the MPS field, there is also the need to monitor tissue stiffness to efficiently mimic the physiologic nature of targeted tissue. For instance, the elasticity of human organs and tissues ranges from a few kilopascals (e.g., liver) to hundreds of megapascals (e.g., tendon) [155]. Cells are sensitive to the molecular cues within their local microenvironment such as matrix stiffness and behave differently in terms of adhesion, migration, and differentiation during normal homeostasis and disease states [156,157]. Moreover, if properly stimulated, cells can tune the stiffness of the tissue from which they belong, in order to generate proper phatophysiologic OoC models. In this view, Liu et al. [55] developed a device to simultaneously stimulate (5% nominal tensile strain at 0.1 Hz, 8 h/day for 15 days) and record strain measurements of 3D human bone marrow-derived MSCs embedded in PEG-norbornene (PEG-NB) hydrogels (Figure 6b). PEG-NB is a biomaterial with tunable elasticity and degradability generally used to mimic soft tissues in the range of a few kilopascals [158]. To estimate tissue stiffness, the authors embedded carbon nanotube (CNT)-based strain sensors into a deformable off-stoichiometry thiol-ene-based PDMS (OSTE-PDMS) membrane and measured its deflection, which is then used as input for a numerical model to calculate the corresponding gel stiffness. In particular, the study shows that when MSCs are mechanically stimulated, they form an α-SMA-rich cell network with a significant production of collagen at day 15. Moreover, the application of 3D stretching stimulation contributed in maintaining the integrity and stiffness of gels (7.95 ± 0.73 kPa at day 15) as compared to static PEG-NB gels where gel softening (2.81 ± 0.78 kPa at day 15) eventually led to tissue degradation. Similarly, Lin et al. [159] fabricated a microfluidic device to study the in-plane elasticity of cellular layers by means of pressure sensors based on electrofluidic circuits. The device is composed of three PDMS layers, namely, cell culture layer, a membrane, and an electrofluidic circuit layer. When the membrane is pressurized, its deformation alters the cross-sectional area of the electrofluidic circuit channel. As a result, the magnitude of the applied pressure can be estimated by measuring the variation of electrical property in the circuit. By adopting this technique, the authors measured the Young’s modulus of myofibroblasts (MRC-5) treated with TGF-β to simulate lung fibrosis, demonstrating an increase in in-plane elasticity from 6.38 to 33.78 MPa when TGF-β was applied. Such experimental results are closer to tissue elasticity values than those obtained through out-of-plane stiffness measurements (e.g., atomic force microscopy).

## 6. Biosensors for Measuring OoC’ Electromechanical Activity

**Figure 7:**
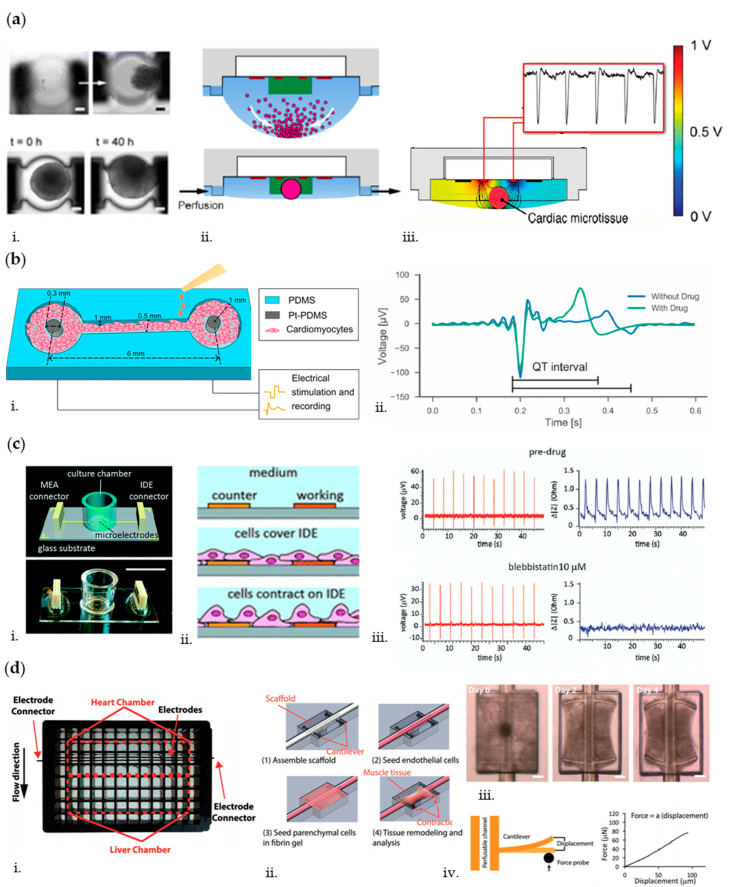
(**a**) Schematic (ii) of on-chip spheroid formation in the hanging drop and subsequent growth monitoring in low-height hanging drops including images at different time points (scale bars are 100 μm, i). Schematic modeling of the tissue-evoked impedance changes in hanging drops network (iii). Adapted from Ref. [163] with permission from ACS Publications. (**b**) Schematic of the 3D Pt-PDMS pillar electrode platform for cardiac tissues (i); 100 nM of the drug isoproterenol was tested on the myocardium indicating clear changes in the QT interval (ii). Adapted from Ref. [52] with permission from Elsevier. (**c**) Schematic and picture of the interdigitated electrodes (IDE)-based chip (i). Scale bar is 1 cm. Schematic of tissue growth and contraction on the IDE (ii) and platform validated with 10 μM blebbistatin: field potentials (MEA, red) and contraction (IDE, blue) data recorded on the same device before and after drug application (iii). Adapted from Ref. [171] with permission from The Royal Society of Chemistry. (**d**) Image of the scaffold multiwall plate including heart chambers (i). A pair of carbon electrodes was embedded into the base plate adjacent to the heart chamber for electrical stimulation. Schematics of the cell seeding process (ii). Brightfield images of the cardiac tissue remodeling process around the scaffold over time (iii). Scale bar, 200 µm. Illustration of the cantilever mechanical test and representative mechanical testing curve showing a linear correlation between cantilever displacement and measured force (iv). Adapted from Ref. [175] with permission from Advanced Functional Materials.

In order to provide a phatophysiologically relevant recapitulation of human organs, OoC systems are required to integrate and control all types of stimulation characterizing the corresponding in vivo situation. As a paradigmatic example, accurate mimicry and simultaneous monitoring of electromechanical cues are crucial for OoC platforms targeting the heart [160]. Indeed, the cardiac tissue is a complex integrated system that leverages electromechanical signals to synchronize cardiomyocytes (CMs) contraction. The correct magnitude, timing, and distribution of these signals are critical for proper functioning of the heart, while aberrant signals can lead to pathological states. Electrical and mechanical stimulations have been reported to enhance cellular maturation, reorganization, and transformation in microfluidic cardiac cell cultures [40,161]. Other than provision of electromechanical stimulation, in vitro assessment of its efficacy is required, calling for the integration of sensors for measurement of electrical signal propagation as well as analysis of contractile forces [162]. To this aim, Schmid et al. [163] developed a PDMS hanging drop network to culture and monitor cardiac spheroids under continuous perfusion (Figure 7a). They adopted an electrical impedance spectroscopy (EIS) technique to measure spheroid growth and to monitor their beating frequency, by placing two 0.5 × 0.2 mm^2^ platinum (Pt) electrodes near the center of each construct. Specifically, EIS is based on the application of an alternating current (AC) voltage to a set of microelectrodes; the resulting current was measured and used to calculate the corresponding interelectrode impedance values [43]. Therefore, the spikes in the EIS signal can be correlated to contractions of the cardiac tissue. A similar technique based on impedance analysis is also reported in a study conducted by Bürgel et al., where, by means of the automated, multiplexed electrical impedance spectroscopy (AMEIS), cardiac spheroids were cultured and monitored in a tilting, high-throughput PDMS device [164]. In their study, they found that contraction of the cardiac spheroids was significantly faster than relaxation, which is consistent with previous observations concerning cardiac spheroids [165].

Disorder in excitation–contraction coupling can contribute to heart failure [166]. At the molecular level, the excitation–contraction coupling is the mechanism responsible to trigger cardiac contraction. In this event, an action potential triggers the calcium-induced-calcium-release process that increases the myoplasmic free Ca^2+^ concentration. The free Ca^2+^ then binds to troponin and activates myosin attachment to the actin filament, pulling the latter to the center of the sarcomere to complete a mechanical contraction [167]. Zhang et al. assessed this phenomenon in their OoC system [168] by means of a high-speed impedance detection technology based on a nonconductive substrate with a pattern of 16 interdigitated electrodes (IDEs) to culture primary neonatal rat cardiomyocytes (NRCM) monolayers and to record the rapid impedance fluctuations induced by their beating [169]. Administration of verapamil, an L-type calcium channel blocker, which reduces calcium influx and action potential duration, resulted in a recorded decrease in the beating rate and contractility of cardiomyocytes, demonstrating the suitability of their heart-on-a-chip to evaluate drug effect. In another study, Zhang et al. [52] developed a PDMS channel for 3D cardiac microtissue culture between two Pt-PDMS pillar electrodes to allow continuous electrical stimulation and impedance recording of local field potentials of cardiomyocytes (Figure 7b). The tissue was electrically stimulated through the Pt-PDMS electrodes by applying trains of bipolar electrical pulses (0.1 mA, 1 ms, 1 Hz, and 30 min/day for 6 days) that are characteristic for native myocardium [170]. After the achievement of mature and stable cardiac tissues, isoproterenol (100 nM), a nonselective β1-adrenergic agonist that is used clinically for the treatment of bradycardia, was used to assess variations of contraction intervals and beating rate consistent with clinical outcomes, making the system suitable for high-quality signal recording.

Cardiac electrophysiology and resulting contractile forces are two distinct and yet correlated functions [167]. A robust platform allowing to measure cardiac electrophysiology and contraction at the same time permits to examine the coupling of electrical and mechanical properties of a heart tissue under the influence of a drug and/or in a disease state. In light of this, Qian et al. [171] developed a device able to culture monolayers of human induced pluripotent stem cells-derived cardiomyocytes (hiPSC-CMs) on top of a glass substrate with patterned microelectrodes for simultaneous real-time mapping of cardiac electrophysiology and recording of contraction. The patterned microelectrodes consisted of (i) multielectrode array (MEA) Pt black-coated electrodes to record field potential (FP) spatial propagation in the cardiac tissue monolayer and (ii) interdigitated electrodes (IDEs) to monitor tissue growth and contraction. As cells grew over the IDEs, the current flow between the working and counter electrodes was impeded in direct correlation to the number of cells covering the electrode and cell morphology [172]. These factors led to changes in the impedance readout (Figure 7c). Furthermore, the signals recorded by means of the high-speed impedance detection method through integrated IDEs enabled to correlate the drug effects on the mechanical contraction of cells. To prove the relevance of monitoring simultaneously electrophysiological and contraction parameters, the authors challenged their system with two well-known compounds: blebbistatin (10 µM), which is used to decrease the contractility of hiPSC-CMs while not altering the electrophysiology of the cells [173], and norepinephrine (400 nM), a clinical drug that increases cardiac beating [174]. Coherently with clinical evidences, blebbistatin led to a flat baseline signal for the impedance recording, while not affecting the action potential, whereas with norepinephrine, the conduction velocity increased by 38% and impedance data showed enhanced spontaneous beating frequency [171].

When a drug is administered in the human body, it enters the vascular systems and diffuse throughout the endothelium to act on target tissues. This systemic condition is often lacking in complex microfluidic systems where the drug is directly administered on target cells without considering the endothelial barrier. To recapitulate this environment, Lai et al. [175] developed a PDMS microwell composed of an endothelialized lumen and two T-shaped microcantilevers embedded with carbon electrodes. This configuration allowed to constrain 3D cardiac microtissue contraction and report forces generated in real-time as well as study the effect of electrical stimulation (Figure 7d). Moreover, this microwell fits a standard 96-well plate format to increase the throughput of the experiments. They used the beam bending theory to noninvasively probe the frequency and force of tissue active and passive contraction. As a demonstration, epinephrine (10 µM) was continuously perfused along the internal vasculature and, as expected, it provoked an immediate increase in tissue contraction frequency and spontaneous beating, while showing no significant increase in contraction force. In another study, Maoz et al. [51] generated an endothelialized myocardium consisting of human umbilical vein endothelial cells (HUVECs) and hiPSC-CMs monolayers. In particular, they developed a transepithelial electrical resistance (TEER)-MEA heart chip to measure the impedance and the integrity of endothelial monolayer as well as to record electrical activity of cardiac cells. The device is a dual-channel PDMS-based platform, consisting of endothelial and cardiac monolayers in the apical and basal channels, respectively. A 0.4 µm pore diameter PET membrane is interposed between channels to allow fluid connections and cellular interactions. The TEER system consists of a 4-point probe configuration (2 electrodes above the endothelial monolayer and 2 below, on the bottom glass substrate hosting cardiac cells), whereas the MEA system is composed of 64 Pt black-coated electrodes patterned on the bottom glass substrate. As validation, they challenged the system with a continuous infusion (60 µL/h) of isoproterenol (50 nM): when the drug was administered in the intact vascular channel (i.e., as monitored by TEER measurement), no effect on cardiac cells was detected, whereas when the endothelium was damaged, the beat rate increased by 28% (i.e., as measured by MEA), coherently with clinical evidences. Their study is a valid model to mimic the systemic vascular drug delivery and demonstrates the advantage of the dual sensor system, which can monitor endothelial barrier function and electrical activity of the cardiomyocytes simultaneously in the same OoC device. 

## 7. Multisensors for Analysis of Multiorgans-on-Chip

As shown in the previous sections, the continuous, on-line monitoring of microenvironmental (i.e., oxygen and cytokines) and physical (i.e., electrical and mechanical) parameters is fundamental to assess the correct behavior and physiological functionality of cell cultures within OoC systems. However, although organs-on-chip platforms are demonstrated to successfully recapitulate human organ functions, they may fail to represent biological processes, which strongly rely on interconnections among different districts of the body. Thus, human multiorgans-on-chip (MOoC) or body-on-chip platforms represent a promising advancement in MPS, as they can recapitulate human organ–organ interactions, as well as drug–drug interplay, which cannot be caught with more simplistic in vitro models. In fact, when a drug is administered in the human body (oral administration), it follows the adsorption, distribution, metabolism, and elimination (ADME) process that needs to be considered when developing or studying new drug compounds. Thus, the development of multiorgan-on-chip devices promises to provide a deeper understanding of interactions between drugs and their metabolites in various organs. Nevertheless, it is crucial to integrate multiple biosensors inside these platforms to monitor the parameters that are relevant for each specific organ.

**Figure 8:**
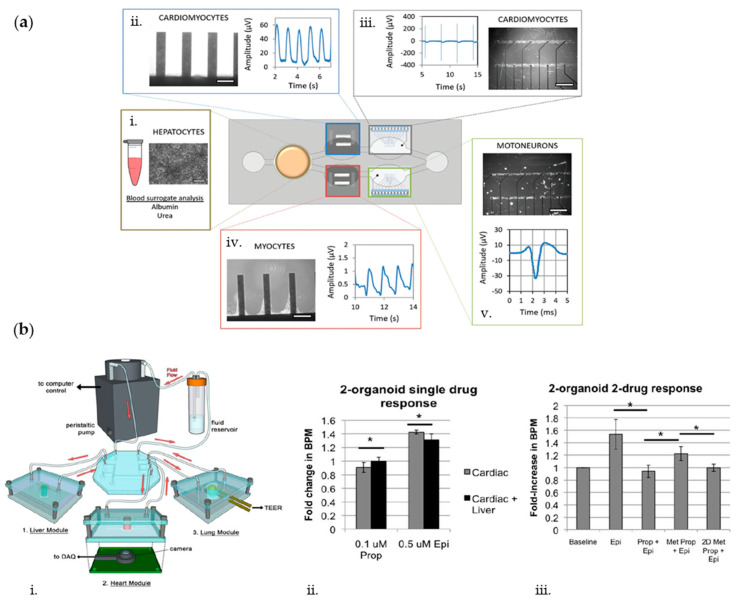
(**a**) Schematic of the pumpless four-organ microfluidic system, showing the acrylic housing holding PDMS gaskets that define the microfluidic pathway and the organ compartments along the path: representative images of hepatocytes on coverslip (i), cardiomyocytes on cantilever chip (CL) (ii) and patterned on customized MEA (cMEA) chips (iii), skeletal muscle myotubes on CL chips (iv), and motoneurons patterned on cMEA chips (v) show each cell type in a different location in the system. Representative functional readouts of the coculture are shown for the different compartments: the supernatant is used to quantify hepatic function (i); the contractile machinery of cardiomyocytes and myotubes is challenged on the CL chips using a laser-deflection based apparatus that records CL movement and a wave amplitude (ii and iv); the electrical signal of cardiomyocytes and motoneurons is recorded from the cMEAs connected to an amplifier via a printed circuit board and an elastomeric connector, translating current changes detected on the electrodes into field potential waveforms (iii and v). Adapted from Ref. [176] with permission from Advanced Functional Materials. (**b**) Schematic of the modular perfusion-driven three-organ-on-a-chip microfluidic system (i): individual microfluidic microreactor units house each organoid or tissue model and are connected via a central fluid routing breadboard. Quantitative analysis of cardiac beating rates (ii) following incorporation of liver organoids show an altered response of the cardiac organoids to both 0.1 μM propranolol and 0.5 μM epinephrine. The assessment of the interplay between the two drugs (iii) shows that BPM (Beats per Minute) values increase from baseline with epinephrine 0.5 μM and increases from epinephrine are blocked by 0.1 μM propranolol in the cardiac organoid-only system. When liver organoids are present and permitted to metabolically inactivate 0.1 μM propranolol, 0.1 μM epinephrine induces an increased BPM value. If 2D-cultured hepatocytes are substituted for the liver organoids, this effect is not observed. Statistical significance: * *p* < 0.05. Adapted from Ref. [178] with permission from Springer Nature.

One example of multiorgan platform was reported by Oleaga et al. [176], which developed a four-organ human microfluidic system able to maintain stable functions of liver, heart, skeletal muscle, and nervous system in continuous communication for 28 days (Figure 8a). Specifically, the microfluidic device is composed of five culture chambers: primary human hepatocytes (PHHs) are cultured and metabolically monitored (via ELISA assays) for urea synthesis, albumin production, and cytochrome P450 activity in the first chamber; induced pluripotent stem cells derived cardiomyocytes (iPSc-CMs) are cultured in two separate chambers, one with customized MEA chips (cMEAs) for electrical stimulation and recordings and one with cantilever chips (CLs) for mechanical contraction monitoring; human skeletal myoblasts are cultured in the fourth chamber (with CLs) for mechanical monitoring; and human motoneurons are cultured in the fifth chamber (with cMEAs) for electrical monitoring. Cardiac and neural electrical activities were measured by arrays of MEA electrodes that translated extracellular current differentials into field potentials. Cardiac and skeletal muscle cells were electrically stimulated, and contractile activities were acquired by translating the amplitude of the CLs movement induced by the contractile cells into mechanical force. Within such system, multiple parameters (i.e., electrical and mechanical properties) could be continuously monitored, pointing out the necessity of developing multisensors systems when dealing with MOoC devices. 

Another example of MOoC is the model developed by Zhang et al. [177], constituted by an automated modular platform encompassing two microbioreactors for the culture of liver and cardiac organoids. Such cultures were in communication via a microfluidics-controlling breadboard for timed routing of fluids and were continuously monitored by optical oxygen sensors and electrochemical immunosensors to measure real-time oxygen concentration and secreted biomarkers levels, respectively. In particular, by means of the immunosensors, they assessed secretion levels of albumin and α-GST from liver organoids and CK-MB from cardiac organoids under APAP and doxorubicin (DOX) treatments. Moreover, thanks to the presence of the oxygen sensor, they could observe that the drug treatment caused no disturbance to the cultures.

As previously stated, body-on-chip devices are particularly appealing in pharmacokinetics (PK)/pharmacodynamics (PD) modelling, as drug responses strongly depend on intertissue interactions. Skardal et al. [178] developed a perfusion-driven MOoC, comprising three modular PDMS platforms for the integration of bioengineered liver, heart, and lung organoids (Figure 8b). Spherical liver organoids and cardiac organoids were formed with human primary cells (hepatocytes, stellate cells, and Kupffer cells), and iPSC-derived cardiomyocytes and primary cardiac fibroblasts, respectively, while lung organoids were developed through layers of cells (lung fibroblasts, epithelial cells, and vascular endothelial cells) over a porous membrane. Upon biofabrication of the organoids in the corresponding platforms, the devices were sealed and connected to the circulatory perfusion system through a central fluid routing breadboard. Fluid was then driven through a microperistaltic pump. Additionally, aiming at monitoring various relevant parameters, the platforms were provided with measurement systems. An onboard camera system was integrated within the heart chip to capture real-time videos and a custom MATLAB software was used to obtain a quantitative description of beating rates. The lung platform was then provided with customized TEER electrodes to monitor the barrier integrity in real-time. Finally, electrochemical sensors operating on the principle of impedance changes caused by antibody/aptamer binding were added to the liver compartment to monitor the concentration of three soluble biomarkers (i.e., albumin, α-GST and creatine kinase). As an initial proof-of-concept, the authors studied the effects of epinephrine and propranolol independently on the cardiac functionality, first in a cardiac-only system, and subsequently in a dual-organoid system with an upstream liver construct. Treatment with propranolol (0.1 µM) resulted in a small (≈10%) decrease in beating rate in the cardiac-only system, while treatment with epinephrine (0.5 µM) resulted in a high (≈40%) increase in beating rate. In the presence of the upstream liver construct, there was no decrease in heart beating rate following propranolol treatment, and epinephrine induced a 30% increase in beating rate, both indicating a mitigation effect of the liver organoid to the drug administration. Next, they assessed the interplay between both drugs in the cardiac-only and dual systems by first administering epinephrine and then propranolol. As expected, in cardiac organoids only, propranolol blocked the effects of epinephrine and no increase in beating rate was detected (as compared to 40% increase of the control where no propranolol was administered). However, in the dual system, a 25% increase in beat rate was observed after the epinephrine was administered, suggesting that the liver module was able to metabolize a certain amount of propranolol. These results clearly demonstrated a contribution of the liver metabolism to the drug effects on the cardiac constructs, thus indicating the strong necessity of integrating multiple tissues inside OoC platforms in drug screening applications.

## 8. Outlook and Conclusions

OoC platforms are powerful preclinical tools holding the promise of effectively reducing, refining, and possibly replacing animal testing in the drug development process, in line with the 3Rs (Replacement, Reduction, and Refinement, *Directive 2010/63/EU*) principles. In this regard, OoC systems represent a factual solution that allows to speed up the development of new compounds (both for efficacy and safety screening), reduces costs of reagents and cells, and mitigates the rising ethical concerns on animal experiments with the advantage of adopting cells of human origin that are better predictive of human-specific clinical outcomes. However, the path of OoC to market is still long, and it is partly dependent on the capability to integrate high-throughput monitoring systems. As we reported, many progresses have been made in the development of on-chip monitoring strategies in OoC. However, while different types of biosensors for continuous and real-time monitoring of intraconstruct environmental (e.g., oxygen and cytokines concentration) and physical (e.g., cardiomyocytes beating properties, barrier integrity) parameters have been studied and adopted in a variety of OoC platforms, their daily application is still hampered by sensors’ specific disadvantages. Optical and electrochemical biosensors are nowadays the preferred choice for real-time monitoring of cellular behaviors (i.e., contraction as well as metabolism), although displaying some limitations, including low-resolution and short sensor lifetime due to electrode saturation. Along with electrochemical sensors, the possibility to miniaturize and parallelize multiple sensors renders electrical impedance spectroscopy, the most straightforward approach, to realize full integration inside standardized impedance-based OoC platforms; however in this case, the main disadvantage is the complexity in data analysis and the high cost of the system. The integration of MEA inside OoC have been proved to allow recording of nervous and cardiac electrical activity with high spatial and temporal resolution, but most of MEAs have been adapted only for 2D models, thus limiting the possibility to monitor 3D constructs. Finally, TEER measurements have been widely adopted to quantify barrier integrity inside OoC, but they suffer from variability due to electrodes positioning, temperature, medium composition, as well as cell culture period. All these factors, together with the intrinsic variability due to the measuring setup, make the comparison among TEER values challenging. An interdisciplinary approach is thus needed to improve the existing types of biosensors, or to develop novel types of biosensing strategies, capable of overcoming all these drawbacks. 

Another major issue regards the inclusion of different types of biosensors within a single device when dealing with MOoC platforms, as each specific tissue needs to be monitored in a proper way. Ideally, upon challenging an OoC with biochemical and/or physical stimuli, it would be desirable to read, in parallel and in each organ compartment, resultant oxidative species, metabolized nutrients, and electromechanical responses. This would allow to gauge not only the reliability of the system setup but also to monitor tissues development, health, and function. Towards this goal, some integrated systems have been proposed and presented above, but considerable improvements are still needed in this field. For example, when modelling the ADME process, the development of connected functional intestine and liver tissues is paramount to study drug candidate’s adsorption and metabolism (i.e., first pass metabolism) in order to detect reliable drug reactions, which would be altered if such tissues were not properly modelled and controlled. Such a platform should necessarily include oxygen sensors to continuously verify that the different tissues sense proper physiological oxygen values, i.e., an aerobic environment for intestinal epithelial cells and liver cells, and an anaerobic environment for microbiome. Furthermore, the platform should be integrated with TEER electrodes, to monitor the integrity of the intestinal epithelium throughout the entire culture period, and real-time albumin and cytochrome P450 detectors, to examine liver functionality and metabolism, respectively. As reviewed in previous paragraphs, such sensors have been so far only integrated into microfluidic devices for end-point analysis, limiting in situ data acquisition that is fundamental for real-time adjustment of culture condition. Thus, one of the future advancements is the development of novel sensors that can be placed inside the culture area to monitor metabolite release (e.g., albumin) directly in situ. It is thus evident that in this case, as well as in most MOoC devices, the major challenges will be the fabrication of multiple sensors having compatible manufacturing techniques, as well as their integration within a single platform, so that they do not interfere with each other. Furthermore, the interface complexity (e.g., large numbers of connections) should be avoided as much as possible, to produce MOoC eventually compatible with standardized platforms formats commonly adopted by biotech and pharmaceutical companies. In this regard, collaborations not only with different disciplines (i.e., genetics, machine learning, and robotics) but also with end-users (e.g., pharmaceutical companies) are needed to design sensors that are capable of automated assessment of MOoC platforms in a real-time, continuous, and high-throughput manner. Once these challenges are addressed, MOoC integrating such next-generation biosensors will represent key tools to comprehensively recapitulate complex and specific pathological conditions, while moving towards an easier acceptance by industrial players. For example, MOoC could be extremely important in studying tumors. The ability to study and monitor the interactions between cancerous tissues and other organs will provide deeper understanding about cancer biology and contribute to progress in drug discovery, biomarker detection, and long-term pharmaceutical metabolism, aiming at improving cancer treatment. Thus, multisensor and multiorgan platforms will be fundamental in the screening process of drugs against cancer and other diseases, eventually replacing animal models in accordance with the 3Rs. As MOoC enable the recapitulation of organ–organ interactions by adopting cells of human origin, problems related to attrition in clinical phases and subsequent drug withdrawal could be substantially reduced. Considering all this, it is expected that in the next future, MOoC integrating multiple biosensors will be widely adopted across research laboratories, clinical applications, and personalized medicine studies
